# Rejuvenescence suggests recurrent coral responses in Miocene and modern reefs near upper thermal tolerance limits

**DOI:** 10.1038/s44185-026-00152-7

**Published:** 2026-08-15

**Authors:** Markus Reuter, Werner E. Piller, Mathias Harzhauser, Francesca R. Bosellini, Fabrizio Lirer, Tayebeh Mohtat, Diego K. Kersting

**Affiliations:** 1 Institute of Geography and Geology, University of Greifswald, Greifswald, Germany; 2 Department of Earth Sciences, NAWI Graz Geocenter, University of Graz, Graz, Austria; 3 Department of Geology and Palaeontology, Natural History Museum Vienna, Vienna, Austria; 4 Department of Chemical and Geological Sciences, University of Modena and Reggio Emilia, Modena, Italy; 5 Department of Earth Sciences, Sapienza University of Rome, Rome, Italy; 6 Geological Survey of Iran, Tehran, Iran; 7 Torre de la Sal Aquaculture Institute, Spanish National Research Council (CSIC), Ribera de Cabanes, Spain

**Keywords:** Climate sciences, Ecology, Ecology, Evolution, Ocean sciences

## Abstract

Understanding coral–climate interactions through deep time is essential for evaluating reef resilience under ongoing climate change. Rejuvenescence is a rare and poorly documented phenomenon in fossil and extant reef-building scleractinian corals, involving polyp contraction followed by regeneration, and has previously been described as a survival strategy for coping with acute thermal stress in a living species. Here, we report evidence of rejuvenescence from a fossil reef in the Makran region of Iran, dated to the Miocene Climatic Optimum, and place it within an ecological and evolutionary framework. Its occurrence in both Miocene Iranian *Acanthastrea* cf. *polygonalis* and modern Mediterranean *Cladocora caespitosa*, despite their phylogenetic, temporal, and biogeographic separation, suggests a recurrent morphogenetic response associated with inferred thermal stress and potentially linked to metabolic downregulation, while its evolutionary origin remains unresolved between deep homology and convergent evolution. This putative role of rejuvenescence in promoting survival appears to be restricted to individual colonies or species and does not translate into thermal resilience at the reef-community scale. Accordingly, current evidence does not support rejuvenescence as a mechanism enhancing coral reef climate resistance but instead identifies it as an indicator of conditions approaching upper thermal tolerance limits in reef ecosystems across geological timescales.

## Introduction

Tropical coral reefs are among the ecosystems most vulnerable to climate change^1,2^. The health and ecological functioning of these highly diverse marine systems depend critically on the symbiosis between reef-building scleractinian corals and their dinoflagellate endosymbionts, which can provide more than 90% of the host’s energy requirements through photosynthesis^3–5^. Thermal stress disturbs this symbiosis, compromising coral fitness and increasing susceptibility to starvation and disease, often culminating in tissue necrosis and mortality^6–8^. Ongoing ocean warming has intensified the frequency and severity of marine heatwaves, driving widespread bleaching and mortality events and threatening the long-term viability of coral reefs worldwide^9,10^. Geological evidence indicates that reef corals persisted through past intervals of elevated global temperatures, suggesting that ancient adaptive traits may underpin the future persistence of shallow-water coral reef ecosystems^11,12^. However, substantial uncertainties remain regarding how ancient corals responded to extreme thermal events and what survival strategies they employed.

Corals can sometimes regenerate after extensive tissue necrosis, even when colonies appear externally dead. Such recovery requires that small patches of living tissue persist to initiate regrowth. These residual tissues may be sheltered deep within the skeletal structure, remaining viable even when the colony surface becomes overgrown by other organisms (corals, coralline algae) or buried by sediment^13–16^. Regeneration from these internal tissue remnants is known as the “phoenix effect”^17^, producing nonspecific growth interruptions. In contrast, rejuvenescence refers to regeneration from minute polyp remnants that survive at the colony surface^7^. First documented in extinct Paleozoic rugose corals, rejuvenescence involves polyp contraction and the formation of a reduced internal corallite nested within the pre-existing skeletal framework^18^. Across the 250-million-year evolutionary history of scleractinian corals, this morphological feature has been described only in Early Jurassic zardinophyllid corals of southern France—an enigmatic early Mesozoic group combining rugose-like corallite characteristics with the aragonitic skeleton typical of modern scleractinians^19^—and, recently, in living *Cladocora caespitosa* of the Mediterranean Sea^7^. The striking morphological similarity between rejuvenescence structures in both fossil and extant taxa suggests an evolutionarily conserved response, possibly related to environmental stress^7^. Whereas sedimentation and food limitation have been proposed as triggers of rejuvenescence in fossil rugose corals^20,21^, marine heatwaves have been the most likely trigger in modern *C. caespitosa*^7^. This observation raises the hypothesis that rejuvenescence may represent a deep-time adaptive response for withstanding episodic thermal stress^7,22^. Nonetheless, its extreme rarity in both fossil and living scleractinians leaves unresolved questions concerning the specificity of its environmental triggers and its broader ecological relevance.

**Fig. 1:**
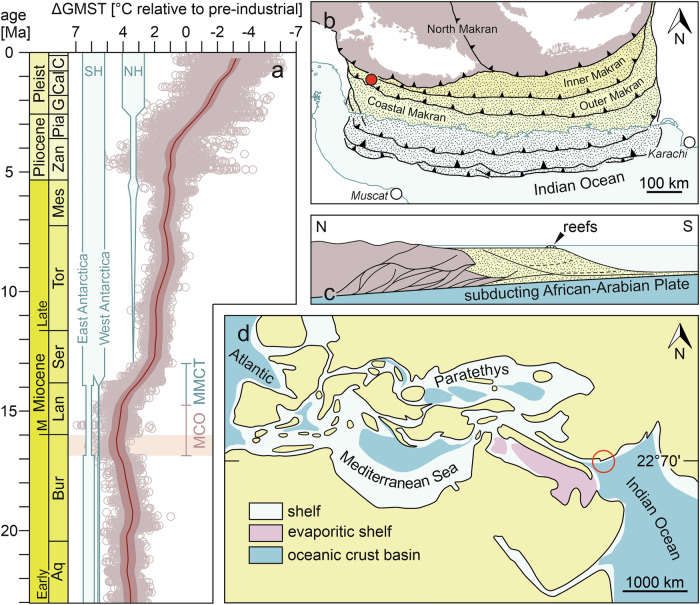
Stratigraphic, geological and paleoclimatic context. **a** Global mean surface temperature (GMST) record; proxy data (open circles) and statistically reconstructed 500-kyr mean values (solid red line) with 95% credible interval, based on data from^90^. Blue vertical bars indicate approximate estimates of ice volume for the southern (SH) and northern (NH) hemispheres after^23^; MCO Miocene climate optimum, MMCT middle Miocene climate transition. The light pink horizontal bar marks the inferred age interval of the studied reef based on planktic foraminifers. **b** Structural map of Makran showing the tectonostratigraphic zones of the onshore accretionary wedge, adapted from^26^ (CC BY-NC-ND 4.0) with permission from the copyright holder. The red dot indicates the study location. **c** Depositional environment of the Burdigalian coral reefs in Makran. A narrow siliciclastic shelf (stippled yellow) migrated southward together with the underlying deforming wedge (purple) from the Oligocene to Middle Miocene (~35 to 15 Ma), adapted from^26^ (CC BY-NC-ND 4.0) with permission from the copyright holder; schematic, not to scale. **d** Paleogeography of the circum-Mediterranean to Middle East region during the Burdigalian (20.5–19 Ma), redrawn from^91^ (CC BY 4.0), showing the location and paleolatitude of the studied fossil reef (red circle).

Here, we report evidence of rejuvenescence in a fossil scleractinian coral from the Early Miocene (late Burdigalian, ~17–16 Ma) of the Makran region, Islamic Republic of Iran. This occurrence coincides with the termination of a prolonged warm climatic interval spanning the Oligocene to Middle Miocene, marking the transition from largely ice-free Eocene hothouse/greenhouse conditions to the bipolar-glaciated icehouse climate of the late Neogene and Quaternary^23^ (Fig. 1a). The studied coral was recovered from a turbid reef on the eastern limb of the Band-e-Chakar Syncline near the village of Irer within the Outer Makran tectono-stratigraphic zone of the Makran accretionary wedge (Irer locality; Fig. 1b)^24–26^. The Makran accretionary wedge developed as a result of subduction of the Arabian Plate beneath the southern Eurasian margin since the Eocene^27^. During the Early to Middle Miocene, coral reefs of the Outer Makran formed locally on a prograding wedge-top siliciclastic shelf^26,28^ (Fig. 1c) at approximately 22.7° N in the northwestern Indian Ocean (Fig. 1d). This depositional setting provides a natural laboratory for investigating coral responses to combined sedimentary and thermal stress regimes. Identifying skeletal features that may be associated with environmental stress in fossil corals could improve the assessment of climate extremes in the geological record.

## Results

### Vertical succession of reef facies

**Fig. 2:**
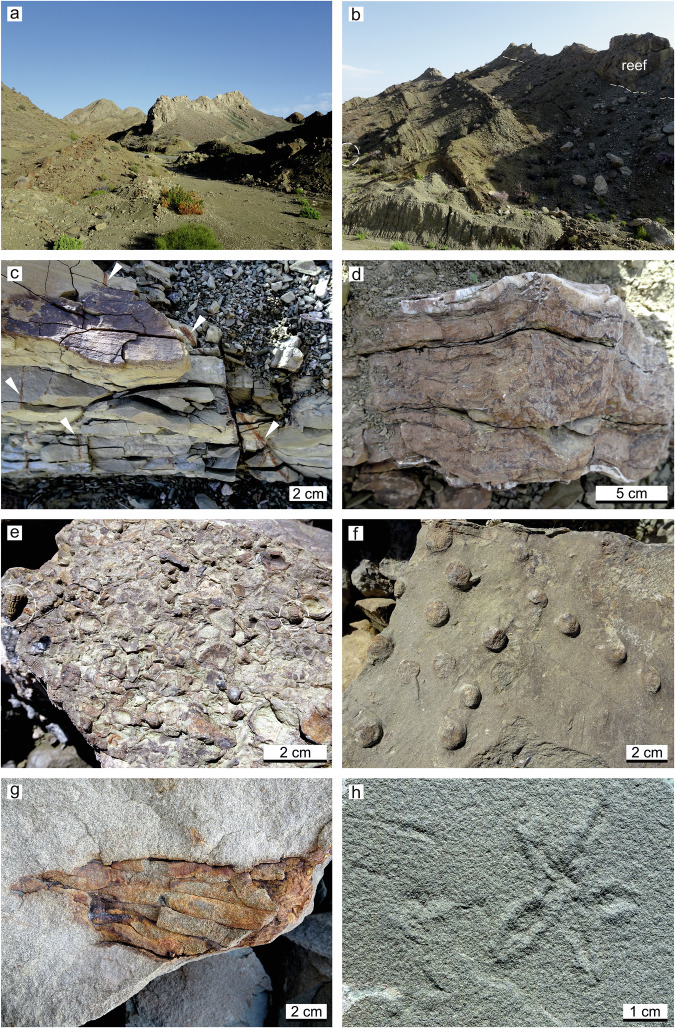
Locality and peritidal facies of the Band-e-Chakar Formation. **a** Tilted sequence of mixed carbonate-siliciclastic shallow-marine deposits at the Irer locality. Erosion leaves resistant reef limestones protruding. View on the reef roof surface and the overlying siliciclastic mudstones and marls (bed 57). **b** Peritidal siliciclastic (sandstone-mudstone) succession below the reef depositional unit. The white dashed line marks the lower boundary of the reef unit. Person for scale (white dashed circle). **c** Vertical fossil rootlets (white arrowheads) confirming colonization of mudflats by vegetation (bed 2). **d** Stromatolithic limestone and fibrous gypsum veins deformed by teepee structures (bed 4). **e** Channel lag coquina containing small oysters, veneroid bivalves, and small gastropods. White dashed circles indicate mangrove-associated potamid gastropods. Disarticulated bivalve shells are orientated in a stable convex-up position by current action in the channel (bed 19). View of the lower surface of the bed. **f** Undertraces of soft-bottom actinian burrows (*Bergaueria*) on a sandstone bed sole (bed 21). **g** Driftwood with *Teredolites* borings (bed 63). **h** Asteroid resting trace (*Asteriacites*) (bed 64). Bed numbers refer to Fig. 3.

**Fig. 3:**
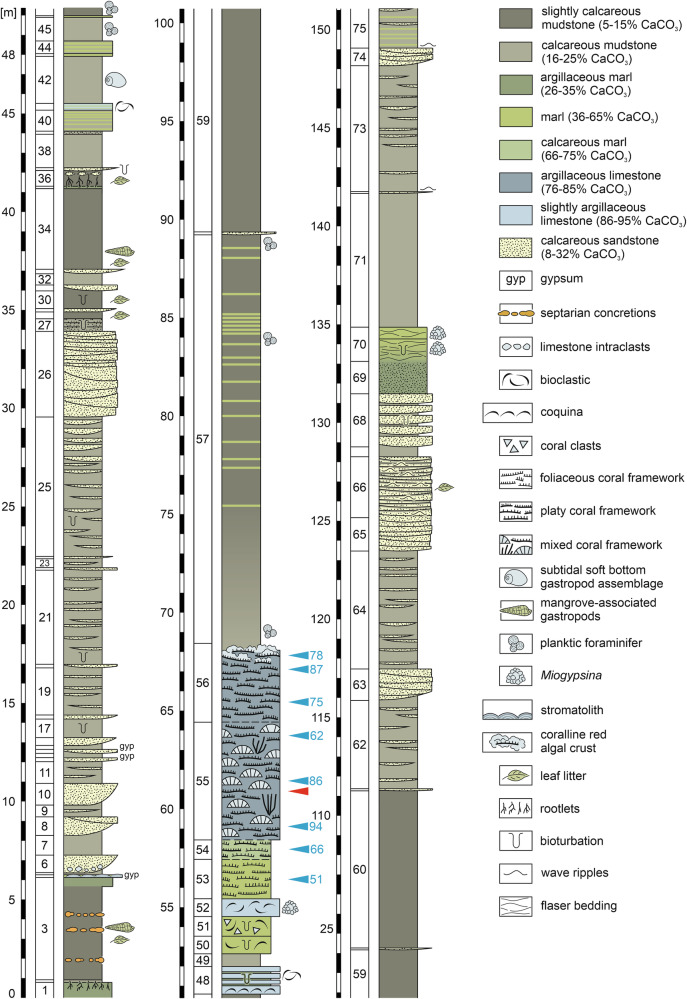
Lithological log of the studied section in the Band-e-Chakar Formation. The stratigraphic position of the coral sample is marked by a red arrowhead. Blue arrowheads show bulk carbonate values (wt%) of the inter-coral matrix. Planktonic foraminiferal symbols indicate the positions of sampled assemblages, as listed in Table 1.

**Fig. 4:**
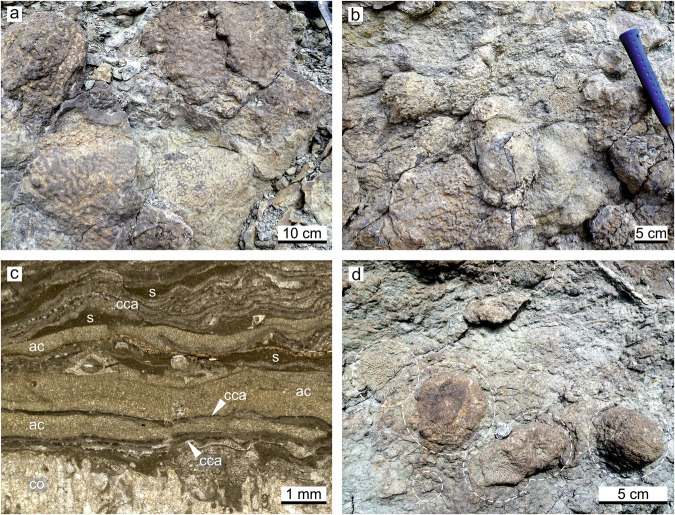
Ecological succession of encrusting organisms. **a** Species-rich assemblage of mutually encrusting corals in the mixed coral facies. Typically, corals from the Irer Reef do not exhibit biogenic encrustation on their surfaces. **b** Only in the uppermost part of the reef are corals extensively covered by biogenic crusts formed by acervulinid foraminifers and crustose coralline algae. **c** Thin-section photomicrograph (longitudinal section) of the biogenic crust carpeting Irer Reef (co coral, ac acervulinid foraminifer, cca crustose coralline alga, s sediment). **d** Active framework accretion ends with the algal-foraminiferal crust. However, the crust continues to serve as substrate for a non-framework solitary coral community (indicated by dashed white circles); **a**, **b**, **d** show the corals in top view.

The studied coral reef (Fig. 2a) and its underlying and overlying sediments belong to the Burdigalian Band-e-Chakar Formation^24,25^. The exposed 13-m-thick reef unit conformably overlies siliciclastic mudstones, sandstones and marls (Fig. 2b), which record a transition from mangrove-fringed tidal flats to shallow subtidal soft bottom environments^25^ (Fig. 2c-f). These deposits are laterally continuous over several hundred meters, without apparent facies changes (Fig. 2b). The investigated coral reef stratigraphic section (Fig. 3) shows a gradual vertical shift in framework composition from (1) a ~3 m thick foliaceous coral framework (hereafter ‘foliaceous coral facies’), to (2) a ~6 m thick mixed framework of massive, platy, and robust branching corals (hereafter ‘mixed coral facies’), and finally to (3) a ~4 m thick platy coral-dominated framework (hereafter ‘platy coral facies’). Foliaceous corals are defined here as thin, undulating, or folded sheet-like forms, typically <1 cm thick, whereas platy corals comprise thicker, relatively flat, horizontal plates on the centimeter scale. No lateral facies changes are observed within the foliaceous, mixed, or platy coral facies exposed in the outcrop. Faunal turnover associated with vertical transitions in dominant coral growth forms cannot be resolved taxonomically because field-based characterization is constrained by the uneven distribution of well-preserved coral surfaces free of lithified sediment and coral overgrowth. This limitation precludes systematic quantitative assessment of coral communities along continuous stratigraphic or spatial transects. Nevertheless, our observations are consistent with those of Ghaedi et al.^24^, who distinguished two coral assemblages in the Irer reef section. The lower platestone assemblage, dominated by agariciid corals including *Pavona* and *Cyathoseris* together with other platy taxa, corresponds to the foliaceous coral facies defined here. In the upper part of the section, Ghaedi et al.^24^ identified a platestone-domestone assemblage characterized by genera such as *Caulastraea*, *Heliastraea*, *Tarbellastraea*, and *Acanthastrea*, which likely corresponds to the mixed and platy coral facies recognized in this study (Fig. 4a).

The carbonate content in the mud-fraction of inter-coral sediment is variable, ranging from 51 to 94 wt% (mean: 75 ± 13 wt%, *n* = 8) (Fig. 3 and Supplementary Data 1). Overall, the fine siliciclastic influence is higher in the lower part of the reef section, i.e., the foliaceous coral facies (Fig. 3). The upper surface of the reef is marked by a centimeter-thick encrustation formed by acervulinid foraminifers (*Solenomeris*), which are volumetrically dominant, and crustose coralline algae (*Mesophyllum* and possibly *Spongites*) (Figs. 3 and 4b, c). Thin mud layers with single detritic quartz grains are interbedded between the biogenic layers (Fig. 4c). The algal-foraminiferal crust covers the in-situ reef corals (Fig. 4b) and serves as a substrate for solitary corals (Fig. 4d). Above this crust, the sedimentary succession continues with planktonic foraminifer-rich siliciclastic mudstones and marls, which gradually grade upwards into peritidal siliciclastic mudstones interbedded with sheet- or patch-like sand deposits and sand dominant channel fills (Figs. 2a, g, h and 3). The peritidal facies is laterally traceable over large areas and shows no discernible internal variations.

The larger benthic foraminifer *Miogypsina globulina* occurs at the base of the coral limestone and within peritidal facies in the upper part of the studied depositional succession (Fig. 3). Planktonic foraminifers are present immediately both below and above the coral limestone (Fig. 3), although preservation is poor and diversity low (Table 1).

**Table 1:** Occurrence of planktonic foraminifers in the studied section

Species	Below reef		Above reef		
	Bed 45, 0.6 m	Bed 47, 0.4 m	Bed 57, 0.25 m	Bed 57, 15.4 m	Bed 57, 20.6 m
*Dentoglobigerina altispira*	x				x
*Dentoglobigerina baroemoenensis*			x	x	
*Dentoglobigerina venezuelana*			x		
*Globigerinoides altiaperturus*			x		
*Globigerinoides subquadratus*	x	x	x		
*Globigerinoides* spp.			x		
*Globoroturborotalita pseudopraebulloides*			x		
*Globoturborotalita woodi*			x		x
*Paragloborotalia acrostoma* (left coiled)			x		x
*Paragloborotalia bella*			x		
*Trilobatus trilobus*	x	x			x

Bed numbers refer to Fig. 3. Meters (m) indicate height above the base of the respective bed.

### Features of coral growth disturbances and recovery

**Fig. 5:**
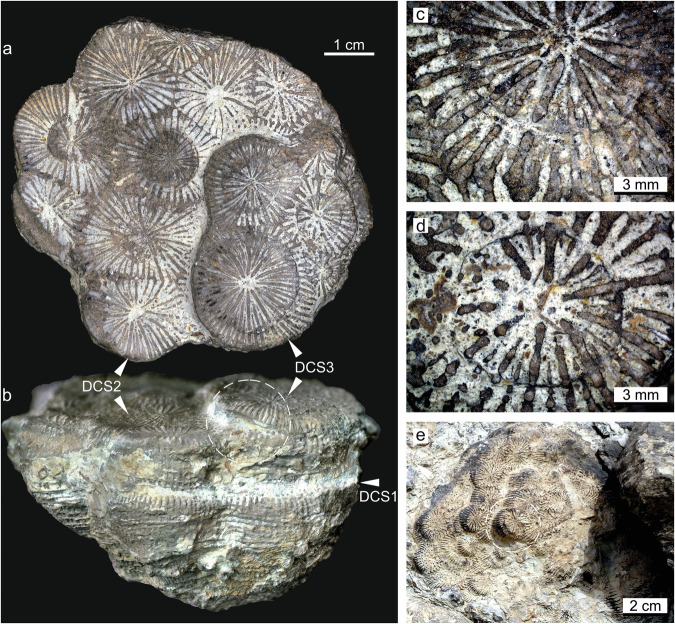
Growth characteristics of rejuvenated *A*. cf. *polygonalis*. **a** Top view of the colony showing double corallites. DCS double corallite surface. **b** Lateral view of the same specimen. The white dashed circle highlights peripheral overgrowth of sediment deposited on DCS2. The narrowing of corallites associated with DCS1 indicates an external rejuvenescence scar^7^. **c**, **d** Detail of double corallites on DCS3. Some septa show continuity inside the new external wall. **e** Another specimen of *A*. cf. *polygonalis* in the mixed coral facies showing possible rejuvenescence features. The white dashed circle points to a corallite with septa extending across a well-developed inner epitheca.

This study focuses on a massive, cerioid corallum of *Acanthastrea* cf. *polygonalis* Felix, 1921 (non Martin, 1880) (Fig. 5a, b). The original skeletal aragonite of this coral is completely transformed into calcite, and the primary pore space is fully cemented, as is the case for all corals in the outcrop. This mode of preservation results in the destruction of the primary density banding and geochemical signals; however, it preserves the surface morphology of fossil corals in partly remarkable detail. The upper surface of the specimen studied is especially notable for the presence of small (inner) corallites positioned at the center of larger (outer) corallites, a configuration referred to hereafter as ‘double corallite’. Outer and inner corallites of a double corallite are separated by a distinct epitheca but share common septa (Fig. 5c, d). The fossil coral displays two distinct surfaces with double corallites (‘double corallite surface’, DCS): the lower one (DCS2) marks a growth hiatus, while the upper one (DCS3) represents the final growth surface (Fig. 5a, b). An earlier growth disturbance (DCS1) is evident in the side view, indicted by a sudden narrowing of corallite diameters, which also suggest the presence of double corallites (Fig. 5b). While the coral completely recovered after DCS1, DCS2 is followed by a constriction of the corallum diameter by about two thirds (Fig. 5a). Above this constriction, the remaining portion of the coral expanded laterally over muddy sediment that covers DCS2 (Fig. 5b).

The vertical distance from the base of the coral colony to DCS1 measures 2.3 cm, with subsequent intervals of 1.0 cm between DCS1 and DCS2, and 0.5 cm between DCS2 and DCS3. Only 22% of the corallites exposed on DCS2 (*n* = 9) show an inner corallite wall, compared to 80% on DCS3 (*n* = 5). On DCS2, the diameter of the inner corallite is reduced by 48.5 ± 8.5% relative to the outer corallite, and on DCS3 by 38.8 ± 12.4%.

Within the studied reef section, double corallites were observed only in *A*. cf. *polygonalis*; no other taxon exhibited this feature. Even in this species, double corallites are rare but not a singular phenomenon. In addition to the specimen described in this study, a second *A*. cf. *polygonalis* colony showing double corallites was identified in a field photograph (Fig. 5e). *A*. cf. *polygonalis* occurs in both the mixed coral facies and the platy coral facies, but is absent from the foliaceous coral facies. Importantly, the specimens with double corallites originate from the mixed coral facies, whereas this feature was not observed in the platy coral facies. However, these field observations could not be systematically quantified due to the low abundance of this species in the outcrop and the limited availability of sediment-free coral surfaces that allow for identification of double corallites.

## Discussion

Despite the limited number of samples and the low abundance of planktonic foraminifers, the assemblage (Table 1) contains several biostratigraphically indicative taxa. The occurrence of *Globigerinoides subquadratus* and *Trilobatus trilobus* in bed 45, although both possessing long stratigraphic ranges, restricts the age to not older than planktonic foraminiferal subzone M1a (Aquitanian)^29,30^. At the base of bed 57, the presence of *G. altiaperturus—*whose first appearance lies in zone M2^29,31^ and last appearance at the top of zone M4 (late Burdigalian)^29,30^—supports assignment to the Burdigalian. The occurrence of the larger benthic foraminifera *Miogypsina globulina* at the base of the coral limestone (bed 52), also reported by Ghaedi et al.^24^, provides an additional important biostratigraphic marker. Although *M. globulina* is generally regarded as ranging from the Aquitanian to the Burdigalian^32,33^, its first appearance in the Indo-Pacific corresponds to the base of planktonic foraminiferal zone N6 (sensu Blow^34^), equivalent to the late Burdigalian, with extinction at the Burdigalian/Langhian boundary^33^. Taken together, the presence of *Miogypsina globulina* immediately below and *G. altiaperturus* directly above the coral limestone (Fig. 3 and Table 1) constrains this stratigraphic interval to the late Burdigalian. This timing corresponds to the initial phase of the Miocene Climatic Optimum (MCO, 16.9–14.7 Ma)^35,36^, placing reef formation at approximately 17 to 16 Ma (Fig. 1a).

The vertical depositional succession, marked by a transition from tidal to coral reef and back to tidal facies (Fig. 3), records a transgressive–regressive cycle superimposed on the large-scale, tectonically driven progradational infill of the Makran Basin during the Miocene^26^ (Fig. 1c). In the study area, extensive tidal-flat deposits in both the underlying and overlying strata, together with laterally uniform reef facies, indicate development on a low-gradient ramp with limited environmental heterogeneity. Under such conditions, even minor sea-level rise would have promoted pronounced landward coastline retreat, increasing the distance to terrigenous sediment sources and reducing siliciclastic input to the reef. The upsection decline in the fine-grained siliciclastic fraction of inter-coral sediments reflects this trend but is punctuated by episodic increases in siliciclastic supply, expressed as variations in inter-coral sediment carbonate content (Fig. 3) and as thin terrigenous layers within the upper algal–foraminiferal crust (Fig. 4c).

Coral morphology is strongly controlled by light availability^37–39^. Foliaceous forms, characterized by high surface-area-to-volume ratios and inclined to curved surfaces that enhance light capture and passive sediment shedding, prevail in the lower, most siliciclastic-influenced interval of the studied reef section (Fig. 3) and are typically associated with turbid, light-limited shallow-water environments^40–43^. In contrast, the overlying mixed coral facies (Fig. 3) consists predominantly of massive corals, with subordinate robust branching forms, consistent with reduced turbidity and increased light penetration^39,44,45^. We therefore interpret the vertical transition from siliciclastic-dominated, foliaceous coral facies to carbonate-dominated, mixed coral facies as reflecting increased ecologically effective photic depth driven primarily by reduced turbidity linked to shoreline migration on a low-gradient ramp, with only minor influence from relative sea-level rise and associated slight deepening.

Upsection, the biological succession from mixed to platy coral facies—dominated by light-efficient platy coral growth forms^39,40^—followed by the development of algal–foraminiferal crust under persistently low terrigenous input, is consistent with diminishing light availability during progressive reef drowning^46^. In line with this interpretation, a slight increase in the abundance and diversity of planktonic foraminifers in the overlying muddy deposits (Table 1) suggests increasing influence of open-ocean conditions. This local drowning event coincides with global sea-level rise during the MCO, linked to partial retreat of Antarctic ice sheets in response to global warming^47^ (Fig. 1a).

The shared septa between the inner and outer corallites of a double corallite (Fig. 5c, d) clearly indicate that they belong to the same polyp and represent the diagnostic feature of polyp rejuvenescence^7^. Accordingly, the double corallites observed in the coral specimen from this study cannot be interpreted as secondary colonization of abandoned corallites. In contrast, the Early Jurassic record of rejuvenescence in the zardinophyllid genus *Cryptosepta*^19^ cannot be confirmed with certainty, as the septa are poorly developed (cryptic). Although aperture retraction observed in transverse section might suggest rejuvenescence, this feature appears instead to be associated with the formation of a new septal apparatus^19^.

DCS2 preserves evidence of two successive partial mortality events (Fig. 5a). The first affected the entire colony surface, leading to widespread polyp contraction, whereas the second impacted only specific areas that became covered by sediment, which was subsequently overgrown by the surviving part of the colony. In modern *C. caespitosa* colonies, most of the heat-affected tissue typically dies, while only a few polyps persist through rejuvenescence^7^. During recovery, the surviving, rejuvenated polyps reproduce asexually and overgrow the dead skeletal portions, even when these are covered by algae, leaving an inner scar within the colony^7^. It is therefore possible that the overgrown sediment on DCS2 accumulated after tissue necrosis on those parts of the colony that had died. However, the presence of double corallites beneath the sediment cover on DCS2 (Fig. 5b) suggests that localized sediment accumulation caused partial mortality, rather than resulting from it, by interrupting the rejuvenescence-mediated recovery process. Recovery appears to have succeeded only in unburied areas, indicating that rejuvenescence was not an effective survival strategy under elevated sedimentation rates. From the sequence of partial mortality and recovery at DCS2, we infer that the coral was intermittently exposed to two distinct types of stress: sedimentation, which constrained colony size through localized mortality, and a separate stressor responsible for inducing polyp contraction across the entire colony.

Evidence from a single extant coral species (*C. caespitosa*) suggests a possible link between rejuvenescence and thermal stress^7^. Thermal stress produces distinct geochemical and growth signatures in both modern and Miocene coral skeletons^48–51^. However, reconstruction of such environment – calcification relationships requires preservation of primary skeletal mineralogy and density, a condition not met in the studied specimen, nor in other fossil corals from the Irer Reef. Thus, although the rejuvenescence features observed in the *A*. cf. *polygonalis* closely resemble those documented in modern *C. caespitosa* exposed to heat stress, alternative drivers cannot be definitely excluded. Resolving the causes of polyp rejuvenation in Miocene corals therefore requires an actualistic assessment of modern rejuvenescence processes evaluated against reconstructed Miocene environmental conditions within their broader paleoclimatic context.

**Fig. 6:**
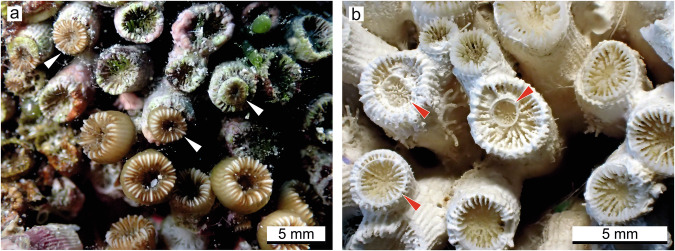
Rejuvenescence in *Cladocora caespitosa*. *C*. *caespitosa* polyps at different stages of rejuvenescence (**a**) and corresponding skeletal structures (**b**) from the Columbretes Islands, Spain (Western Mediterranean Sea). White arrowheads indicate rejuvenated polyps. Some septa remain continuous across the rejuvenation-derived wall (red arrowheads).

Mass mortality events affecting mixotrophic *C. caespitosa* colonies have been attributed to thermally induced starvation^52^. Prolonged exposure to anomalously warm summer conditions destabilizes symbiotic nutrient cycling long before bleaching becomes apparent^53^. In addition, warming enhances stratification in the Mediterranean Sea, limiting vertical mixing and the exchange of surface waters with deeper, nutrient-rich layers, which results in nutrient depletion in the upper water column^54^. This nutrient scarcity induces a state of ‘summer dormancy’ in many benthic suspension feeders^54^, while corals are unable to compensate for the energy deficit caused by disturbed photosymbiosis through increased heterotrophic feeding. This is reflected by the observation that warming-impacted *C. caespitosa* populations exposed to higher nutrient concentrations experience significantly lower levels of mortality^55^. Rejuvenated polyps of *C. caespitosa* (Fig. 6) have been shown to survive heat-induced starvation by reducing their metabolic activity in correlation with their size^7^. In this reduced state, they can persist in metabolic stasis and later regain normal polyp size, enabling regeneration of necrotic colony areas via asexual reproduction once environmental conditions improve^7^.

The Early to Middle Miocene was a period of global warmth relative to today^23^ (Fig. 1a), characterized by elevated tropical sea surface temperatures (SSTs) extending into mid- to high latitudes^56–59^. Within this prolonged warm phase, climatic variability was nevertheless pronounced, driven by orbital forcing and modulated by Antarctic ice-sheet instability^60^. The MCO represents the thermal maximum of this interval, with mean annual global temperatures reaching ~3-4 °C above present^23^ (Fig. 1a). These conditions facilitated the northernmost expansion of coral reef distributions over the last 23 million years^61^. Geochemical sclerochronology and calcification data from exceptionally well-preserved *Porites* fossils from the subtropical Central Paratethys indicate that summer heat extremes during the MCO imposed substantial thermal stress on corals, potentially inducing bleaching even in high-latitude (~45-50° N) marginal reef systems^51^. At lower latitudes, the northwestern Indian Ocean (Arabian Sea) experienced elevated SSTs of ~33 °C during the MCO, based on Mg/Ca records from surface-dwelling planktonic foraminifers^62^ and TEX_86_-derived SST estimates^63^. Relative to the widely accepted optimum temperature range for modern coral reef formation of 23-29 °C^64^, such conditions may have approached or exceeded thermal stress thresholds in shallow reef environments. Direct local SST reconstructions based on geochemical proxy data from planktonic foraminifers sampled from stratigraphic intervals below and above the Irer Reef (Table 1) are not feasible due to extensive diagenetic alteration.

Turbid water tropical reefs in Makran provided a fundamentally different stress environment for reef-building corals than the oligotrophic, clear-water conditions of the modern Mediterranean Sea. Although high concentrations of suspended sediments can adversely affect corals through effects such as light limitation, polyp smothering, tissue abrasion, and reduced reproductive success^65–67^, they can also mitigate light stress by reducing irradiance during thermal anomalies, potentially preventing bleaching^68–71^. Furthermore, turbid reef environments are enriched in dissolved and particulate organic matter, including zooplankton and its derivatives, which serve as important heterotrophic food sources for corals, buffering against starvation when photosymbiosis is impaired by thermal stress^55,72–74^. It is therefore proposed that turbid reefs may have acted as climatic refugia, enhancing equatorial habitability for reef species during geological warm periods^10,75,76^.

**Fig. 7:**
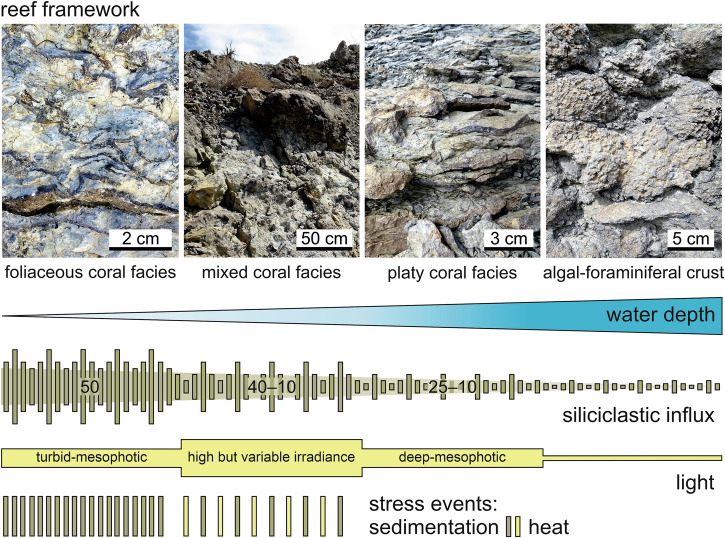
Generalized model illustrating the sequence of sedimentary and paleoecological changes resulting from coral reef drowning. Siliciclastic influx values represent approximate weight percentages (wt%) inferred from carbonate measurements.

At the Irer locality, the restriction of double corallites within a single taxon to the mixed coral facies suggests that specific environmental conditions triggered the rejuvenescence response. The absence of *A*. cf. *polygonalis* in the foliaceous coral facies may reflect its sensitivity to heavily sediment-impacted conditions, as this facies contains the highest siliciclastic content within the vertical reef facies succession (Fig. 3). The overall reduction in fine-grained terrigenous input across the transition from foliaceous to mixed coral facies (Fig. 3) likely alleviated turbidity and sediment deposition rates (Fig. 7), thereby facilitating colonization of the reef by *A*. cf. *polygonalis*. Reduced turbidity is associated with increased exposure of corals to irradiance and temperature stress^77^, which has been proposed as a trigger in modern *Cladocora*^7^ and is consistent with the presence of rejuvenescence features in the mixed coral facies. Fluctuations in the matrix carbonate content (Fig. 3) further indicate short-term variability in siliciclastic input, superimposed on the long-term decline in sediment supply (Fig. 7). In line with this, not all *A*. cf. *polygonalis* colonies in the mixed coral facies show rejuvenescence responses, and the specimen described here may have experienced alternating episodes of thermal and sedimentation stress (Fig. 7). This interpretation is, however, constrained by potential inter-individual variability, as observed in modern, warming-impacted *C. caespitosa* populations from the Columbretes Archipelago, where only 38% of colonies exhibit a rejuvenescence response^7^. In Mediterranean *Cladocora* reefs, rejuvenated polyps can also be easily overlooked in partially necrosed colonies, and affected colonies may regain a macroscopically normal appearance within relatively short timescales (years) following recovery^7^, making the phenomenon cryptic unless the corals are observed at high temporal resolution. In fossil assemblages, features associated with rejuvenescence are therefore most likely preserved only where recovery remained incomplete, introducing a taphonomic bias. Consequently, the apparent rarity of both modern and fossil records likely reflects, at least in part, a combination of biological transience and preservation filtering rather than true ecological scarcity.

Assuming a constant linear extension rate, the decreasing vertical spacing between partial mortality surfaces associated with rejuvenated corallites (Fig. 5b) may indicate an increasing frequency of triggering stress events. The progressively reduced recovery success of the colony—ranging from complete recovery after DCS1, partial recovery after DCS2, to incomplete recovery and ultimate colony death—is consistent with cumulative stress effects^78^. The absence of rejuvenescence in *A*. cf. *polygonalis* within the deeper-water platy coral facies may further suggest that reef drowning reduced exposure to thermal stress during periods of elevated SSTs by lowering irradiance and temperature^79^ (Fig. 7).

Both rejuvenated corals from the Irer Reef belong to the same species, which may indicate a species-specific thermal response in the absence of a broader assemblage-level signal^80^. Such differential sensitivity is consistent with inferred diversification dynamics in tropical shallow-marine systems of the Central Indo-West Pacific from the Oligocene to the Middle Miocene. This interval, spanning the transition from Eocene greenhouse to late Neogene-Quaternary icehouse conditions, was characterized by faunal turnover and low net diversification, suggesting combined effects of elevated extinction and speciation rates^81^. The persistence of thermophilic Eocene relict taxa alongside newly emerging lineages adapted to progressive Cenozoic cooling may have generated reef communities composed of taxa with contrasting thermal tolerances^81^. In this context, tropical turbid reef environments have been proposed as potential refugia and reservoirs of coral diversity, facilitating lineage persistence and subsequent expansion into clearer-water habitats during long-term global cooling^42,82^.

Modern bleaching susceptibility reported in *Acanthastrea* from the Great Barrier Reef^83^, together with the Early Miocene record of rejuvenescence, may suggest a long-standing sensitivity to thermal stress in members of this morphological group. The late Oligocene first appearance of morphologically assigned *Acanthastrea* (Paleobiology Database, https://paleobiodb.org, last access 02-05-2026), with the oldest reported occurrence in the Qom Basin of central Iran^84^, coincides with the Oligocene – Miocene climatic transition. Although this temporal coincidence raises the possibility that the broader trend of Cenozoic cooling provided an environmental context that may have shaped the evolution of thermal sensitivity and associated ecophysiological stress responses in these corals, this interpretation requires caution. Molecular phylogenetic evidence indicates that morphologically defined *Acanthastrea* is polyphyletic within Lobophylliidae^85^, implying that inferred ecophysiological traits may not derive from a single evolutionary origin but instead reflect convergence or clade-specific patterns within the family. The presence of rejuvenescence in members of Cladocoridae (*Cladocora*)^86^ and Lobophylliidae (*Acanthastrea*)^85^ indicates that this response is not restricted to a single phylogenetic lineage. Together with the early divergence of these lineages during the radiation of modern scleractinian corals^87^ and the Jurassic fossil record of *Cladocora* (~201–143 Ma)^88^, this pattern highlights the occurrence of rejuvenescence across coral lineages with long and independent evolutionary histories. Reports from Rugosa^18,20,21^ and possibly zardinophyllid corals^19^, although the identification of these structures as rejuvenescence, their functional interpretation, and their phylogenetic relationships to scleractinian corals remain uncertain, further suggest that comparable stress-related morphogenetic responses may extend deeper into coral evolutionary history. At present, however, available evidence cannot distinguish whether the phylogenetic distribution of rejuvenescence reflects retention of an ancestral stress-response capacity or repeated independent evolution of the capacity to produce comparable morphogenetic responses. Rejuvenescence, involving polyp contraction and skeletal reorganization, is therefore best interpreted as a recurrent stress-associated morphogenetic response: a physiologically mediated process expressed under acute energy limitation that produces coordinated changes in polyp and corallite morphology.

Because rejuvenescence is cryptic and transient, it may be underestimated in both modern and ancient reef systems. Even if more widespread than available records suggest, its functional significance appears limited to individual colonies or species, and present evidence does not support a role for rejuvenescence as a mechanism for enhancing resilience at the reef-community scale. If, as inferred here, rejuvenescence is associated with elevated temperature, it may serve as an indicator of physiological stress as corals approach their upper thermal tolerance limits, providing a rare morphological archive of coral–climate interactions through geological time and offering a long-term perspective on the physiological constraints relevant to reef resilience under ongoing climate change.

## Methods

### Lithological logging and microfacies analysis

A ~150-m-thick lithological section in the Band-e-Chakar Formation was logged bed-by-bed. For microfacies analysis, 25 thin sections (5 × 5 cm) were prepared from solid lithologies (sandstones and limestones). In addition, 15 soft sediment samples (mudstones and marls) were disaggregated in 10% hydrogen peroxide and wet-sieved using 63 and 125 µm mesh sizes for a qualitative assessment of the microfauna.

### Bulk carbonate analysis

A total of 39 bulk sediment samples were powdered and analyzed for total carbon (TC) and total organic carbon (TOC) at the Institute for Earth Sciences, University of Graz (Austria), using a LECO CS-300 analyzer. TOC was determined after acidification to remove carbonates, and total inorganic carbon (TIC) was calculated as the difference between TC and TOC. TIC values were used to estimate bulk carbonate content (calcite equivalent, wt%) using the equation:

1 $${{CaCO}}_{3}=8.34\times {TIC}$$

TC and TOC were each measured in three subsamples, and mean values were used for TIC and CaCO_3_ calculations to minimize the effect of sediment heterogeneity (Supplementary Data 1). For the same reason, only fine-grained sediment was sampled for bulk carbonate analysis, avoiding macroscopic shell and lithic particles.

### Paleolatitude reconstruction and specimen documentation

The paleolatitude of the studied reef was reconstructed using GPlates (https://portal.gplates.org, last access 14-04-2025) based on the PALEOMAP PaleoAtlas for GPlates^89^. The reconstruction assumed a mean Burdigalian age of 18.205 Ma and the present-day GPS coordinates of the studied outcrop (26.68202° N, 57.93381° E), yielding a paleolatitude of 22.7° N. The specimen of *A.* cf. *polygonalis* described herein was imaged and measured using a Keyence VHX-7000 digital microscope. It is deposited in the Geological and Paleontological Collection of the Natural History Museum Vienna, Austria (NHMW 2025/0347/0001).

## Supplementary information

Supplementary Information

Supplementary Data 1

## Data Availability

All data generated for this study are included in this published article and its Supplementary Data file.
