# Supplementary material for: Rejuvenescence suggests recurrent coral responses in Miocene and modern reefs near upper thermal tolerance limits
Source: NPJ Biodivers. 2026 Aug 15;5:26. doi: 10.1038/s44185-026-00152-7 (PMC13476168; doi:10.1038/s44185-026-00152-7)
Supplement: Supplementary file 1 [file 44185_2026_152_MOESM1_ESM.pdf]

Description of Additional Supplementary Files

File name: Supplementary Data 1

Description: Results of Total Organic Carbon (TOC) and Total Inorganic Carbon (TIC) analyses used to determine carbonate concentrations in sediment samples.
